# P-636. Medical Need for Meningococcal Vaccination in Young Children from the Americas

**DOI:** 10.1093/ofid/ofae631.833

**Published:** 2025-01-29

**Authors:** Gaurav Mathur, Maria Gabriela Graña, Reena Ladak, Joanne M Langley, Oluwatosin Olaiya, Alysa Pompeo, Laura Taddei, Rodolfo Villena

**Affiliations:** GSK, Philadelphia, Pennsylvania, USA, Philadelphia, Pennsylvania; GSK, Santiago, Region Metropolitana, Chile; GSK, Philadelphia, Pennsylvania, USA, Philadelphia, Pennsylvania; Dalhousie University, IWK Health and Nova Scotia Health, Halifax, Canada, Halifax, Nova Scotia, Canada; GSK, Santiago, Region Metropolitana, Chile; GSK, Santiago, Region Metropolitana, Chile; GSK, Santiago, Region Metropolitana, Chile; Hospital Dr. Exequiel González Cortés, Faculty of Medicine, Universidad de Chile, Santiago, Region Metropolitana, Chile

## Abstract

**Background:**

Invasive meningococcal disease (IMD), caused mainly by *Neisseria meningitidis* serogroups (Men) A, B, C, W, X, and Y, is an uncommon but serious condition that can lead to life-long sequelae and is fatal in up to 20% of cases even with treatment. IMD incidence is highest in children <5 years. We present the current epidemiology of IMD in the Americas and unmet needs to reduce IMD burden in young children.

**Figure:**
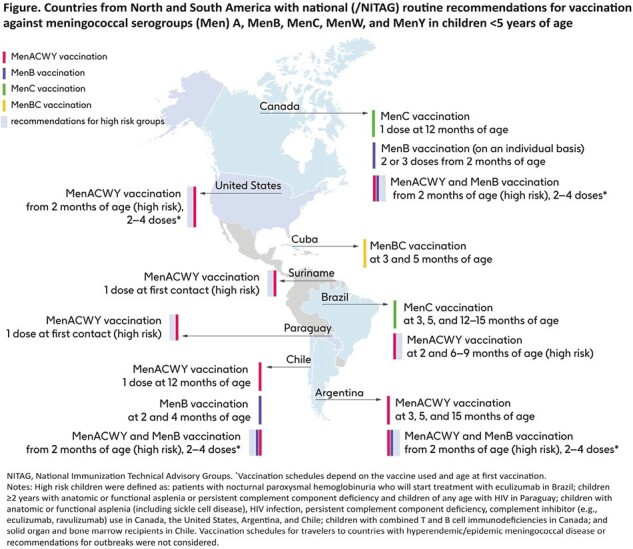

**Methods:**

We reviewed the literature and available surveillance data from 2015 to 2024 to evaluate the IMD burden and national vaccination strategies for children < 5 years of age in the Americas.

**Results:**

Overall, across countries, the incidence is highest in children aged <1 year, followed by children 1–4 years of age. MenB is the predominant serogroup in children < 5 years. In most countries with available data, IMD incidence decreased sharply during the COVID-19 pandemic but increased again after nonpharmaceutical interventions were lifted. Most countries do not have recommendations for routine meningococcal vaccination in children <5 years (Figure). Chile is the only country in the Americas that has routine vaccination against 5 serogroups, by including both MenACWY (2014) and MenB (2023) vaccinations in its national immunization program (NIP). The Argentinian, Brazilian, and Cuban NIPs include MenACWY (2017), MenC (2010), and MenBC (1991) vaccinations in infants, respectively. In Canada, MenC vaccination has been recommended for all infants since 2002, with one province (Manitoba) introducing MenACWY in 2024; MenB vaccination is recommended on an individual basis. Following introduction in the NIP/national recommendations, the incidence of IMD caused by serogroups covered by the vaccines has decreased in these countries. In the United States, MenACWY vaccination is recommended in children at high risk of IMD, but not for routine vaccination in <5-year olds.

**Conclusion:**

Considering the current incidence and burden of IMD in infants < 1 and children < 5 years of age across the region, especially MenB-IMD, comprehensive IMD vaccination programs could reduce the overall burden in this population. NIPs/national recommendations would facilitate equitable access to protection against IMD, aligned to the World Health Organization roadmap to defeat meningitis by 2030.

**Funding:** GSK

**Disclosures:**

**Gaurav Mathur, MD**, GSK: Employee|GSK: Stocks/Bonds (Private Company)|OpenHealth: Writing support **Maria Gabriela Graña, MD**, GSK: employment|GSK: Stocks/Bonds (Private Company) **Reena Ladak, MS**, GSK: Employee|GSK: Stocks/Bonds (Private Company) **Joanne M. Langley, MD**, GSK: Grant/Research Support|Inventprise: Grant/Research Support|Merck: Grant/Research Support|Moderna: Grant/Research Support|Pfizer: Grant/Research Support|VBI: Grant/Research Support|VIDO: Grant/Research Support **Oluwatosin Olaiya, MBChB, MSc**, GSK: employment (current)|Merck: Previous employer **Alysa Pompeo, BPharm**, GSK: employment **Laura Taddei, M.Sc**, GSK: GSK employment|GSK: Stocks/Bonds (Public Company) **Rodolfo Villena, MD**, GSK: Advisor/Consultant|Pfizer: Advisor/Consultant|Pfizer: Grant/Research Support

